# Evolutionary Contribution of Duplicated Genes to Genome Evolution in the Ginseng Species Complex

**DOI:** 10.1093/gbe/evab051

**Published:** 2021-03-13

**Authors:** Ming-Rui Li, Ning Ding, Tianyuan Lu, Jing Zhao, Zhen-Hui Wang, Peng Jiang, Si-Tong Liu, Xin-Feng Wang, Bao Liu, Lin-Feng Li

**Affiliations:** 1 Ministry of Education Key Laboratory for Biodiversity Science and Ecological Engineering, School of Life Sciences, Fudan University, Shanghai, China; 2 McGill University and Genome Quebec Innovation Center, Montreal, Quebec, Canada; 3 Key Laboratory of Molecular Epigenetics of the Ministry of Education (MOE), Northeast Normal University, Changchun, China; 4 Faculty of Agronomy, Jilin Agricultural University, Changchun, China; 5 School of Life Sciences, Jilin University, Changchun, China

**Keywords:** gene duplication model, polyploidy, DNA methylation, gene expression, Panax

## Abstract

Genes duplicated by whole genome duplication (WGD) and small-scale duplication (SSD) have played important roles in adaptive evolution of all flowering plants. However, it still remains underinvestigated how the distinct models of duplication events and their contending evolutionary patterns have shaped the genome and epigenomes of extant plant species. In this study, we investigated the contribution of the WGD- and SSD-derived duplicate genes to the genome evolution of one diploid and three closely related allotetraploid *Panax* species based on genome, methylome, and proteome data sets. Our genome-wide comparative analyses revealed that although the ginseng species complex was recently diverged, they have evolved distinct overall patterns of nucleotide variation, cytosine methylation, and protein-level expression. In particular, genetic and epigenetic asymmetries observed in the recent WGD-derived genes are largely consistent across the ginseng species complex. In addition, our results revealed that gene duplicates generated by ancient WGD and SSD mechanisms exhibited distinct evolutionary patterns. We found the ancient WGD-derived genes (i.e., ancient collinear gene) are genetically more conserved and hypomethylated at the cytosine sites. In contrast, some of the SSD-derived genes (i.e., dispersal duplicated gene) showed hypermethylation and high variance in nucleotide variation pattern. Functional enrichment analyses of the duplicated genes indicated that adaptation-related traits (i.e., photosynthesis) created during the distant ancient WGDs are further strengthened by both the more recent WGD and SSD. Together, our findings suggest that different types of duplicated genes may have played distinct but relaying evolutionary roles in the polyploidization and speciation processes in the ginseng species complex.

**Significance**

Genes duplicated by whole genome duplication (WGD) and small-scale duplication (SSD) mechanisms occurred frequently in the long-term evolutionary process of all extant flowering plants. This study investigated the contribution of duplicated genes derived from WGD and SSD to genome evolution in the ginseng species complex. Comparative analyses revealed that gene duplicates generated by the two duplication models exhibited distinct evolutionary dynamics. Functional enrichment analyses of the duplicated genes revealed that adaptation-related traits created during the distant ancient WGDs are further strengthened by both the more recent WGD and SSD. Our findings suggest that different types of duplicated genes may have played distinct but relaying evolutionary roles in the polyploidization and speciation processes in the ginseng species complex.

## Introduction

Polyploidy or whole genome duplication (WGD) is a ubiquitous feature in all angiosperm lineages (Van de Peer et al. 2009; Soltis et al. 2014; Van de Peer et al. 2017). An estimated 30–70% of extant flowering plants are neopolyploids (Masterson 1994; Wolfe 2001; Wood et al. 2009). The transformative effect of WGD in plant evolution has been recognized as early as one century ago (Lutz 1907; Winge 1917). Yet, opposing opinion still holds which asserts that evolutionarily polyploidy is either noise or a dead-end with little long-term evolutionary contributions (Stebbins 1950; Wagner 1970; Stebbins 1971; Mayrose et al. 2011). In the last decades, increasing ecological and molecular evidence has revealed the critical roles of polyploidy in plant evolution and diversification (Levin 1983; Wood et al. 2009; Landis et al. 2018).

All extant angiosperm plants have experienced at least one round of WGD predating their origin and divergence (Jiao et al. 2011). A striking example of paleopolyploidization is cotton, which has experienced an aggregate 144× multiplication through five times of WGD (2 × 2 × 3 × 6 × 2) spanning from >140 to 1–2 Ma (Paterson et al. 2012; Wendel 2015). However, the overall protein-coding genes annotated in both the modern diploid (40,976–41,330) and tetraploid (70,199–71,297) cotton species are only 1.8–3.1 times compared with the total genes of the ancestral angiosperm genome (22,899) (Wang K, Wang Z, et al. 2012; Li et al. 2015; Murat et al. 2017; Wang et al. 2019). This phenomenon raises a question as to why the duplicated genes are not all retained or lost in extant plant genome after the polyploidization–diploidization cycles. Multiple hypotheses have been proposed to explain the mechanisms underlying differential gene retention, such as gene pseudogenization, subfunctionalization, and neofunctionalization (Panchy et al. 2016; Cheng et al. 2018). In soybean, for example, majority of the gene duplicates show either expression subfunctionalization or neofunctionalization (Roulin et al. 2013). Similarly, comparative epigenomic survey of the duplicated gene in soybean and common bean has further documented the important roles of differential cytosine methylation in determining the patterns of gene expression and evolutionary rate of the gene duplicates (Do Kim et al. 2015). In addition, large-scale assessment of 141 plant genomes also suggested that gene duplicates derived from distinct SSD modes tend to show different evolutionary dynamics and functional features (Qiao et al. 2019). These attributes suggest that the duplicated genes provide a continuous supply of raw materials, and diverse mechanisms have acted together to shape the genetic and epigenetic dynamics of duplicated genes in the short- and long-term evolutionary processes.

**Fig. 1:**
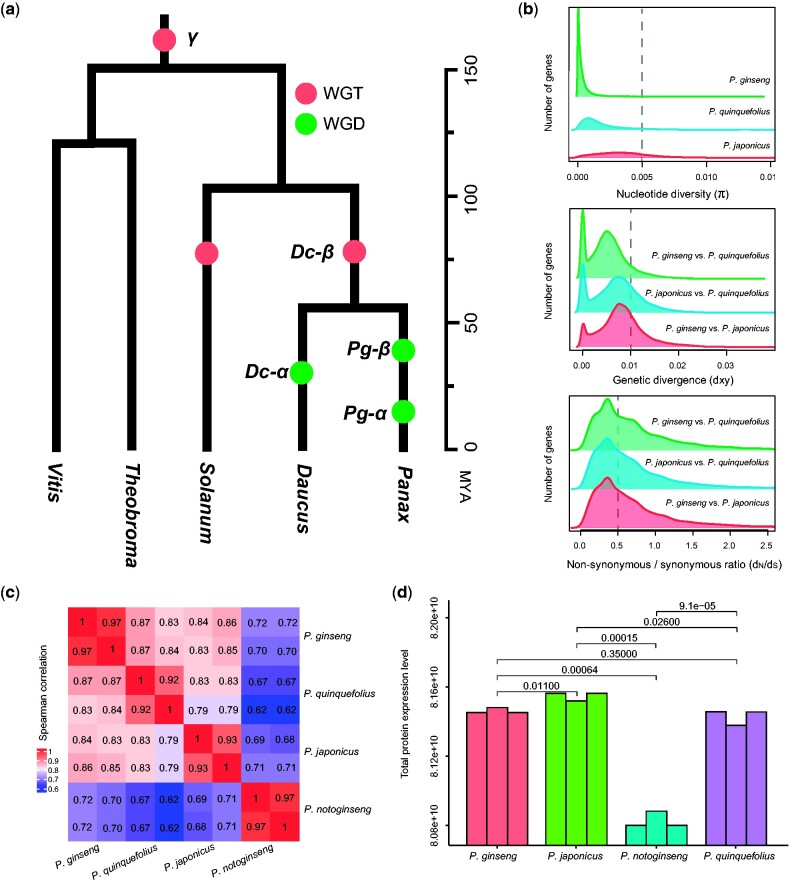
Overall patterns of the four *Panax* species at the nucleotide variation, cytosine methylation, and protein expression levels. (*a*) Species tree and whole genome duplication history of the *Panax* and other selected eudicot species; (*b*) Distribution patterns of the nucleotide diversity (π), absolute genetic divergence (dxy), and selection pressure (*d*_N_/*d*_S_) among the three tetraploid species; (*c*) Correlations of the cytosine methylation level of the four *Panax* species at the intra- and interspecific levels; (*d*) Overall protein expression level of the four *Panax* species based on generalized linear model.

The genus *Panax* includes four well-recognized diploids (2*n* = 2*x* = 24), three closely related tetraploids (2*n* = 4*x* = 48), and a polyphyletic group containing more than five *Panax bipinnatifidus* varieties (Zuo et al. 2011; Shi et al. 2015; Zuo et al. 2017). It has been documented that the genus *Panax* has experienced 36-fold duplication of ancestral eudicot genome through two paleotriplications (γ and Dc-β) followed by two more recent duplication events (Pg-α and Pg-β) (fig. 1*a*) (Choi et al. 2013; Zhang et al. 2017). The γ and Dc-β paleotriplication events have been identified in the common ancestor of all extant Apiales species (Murat et al. 2017; Kim et al. 2018). In contrast, the ancient duplication event Pg-β (24.6–32.8 Ma) is supposed to have emerged predating the diversification of extant *Panax* species (Choi et al. 2013; Zhang et al. 2017). Then, a more recent WGD (Pg-α) (1.6–3.3 Ma) has led to the establishment of three geographically isolated tetraploid species: *Panax ginseng*, *Panax quinquefolius*, and *Panax japonicus* (referred to as ginseng species complex) (Yi et al. 2004; Shi et al. 2015; Zuo et al. 2017). Clear evolutionary trajectories of the ancient and recent WGDs together with available assembled reference genomes of the diploid species *Panax notoginseng* (Zhang et al. 2017) and tetraploid species *P. ginseng* (Choi et al. 2013; Zhang et al. 2017) render the ginseng species complex a suitable system to elucidate the evolutionary contribution of duplicated genes following polyploidy and speciation.

In this study, we focused specifically on how the gene duplicates generated at different evolutionary stages (ancient vs. recent) and by distinct mechanisms (WGD vs. SSD) have shaped the genome evolution of ginseng species complex. To this end, we surveyed the genome, methylome, and proteome of the ginseng species complex and its diploid relative *P*. *notoginseng*. To further evaluate the evolutionary roles of paleopolyploidization (i.e., Pg-β and earlier WGDs) and SSD mechanism, we identified ancient WGD- and SSD-derived genes from the *P. ginseng* and *P. notoginseng* genome. The aims of this study were to 1) evaluate how the duplicate genes derived from WGD and SSD mechanisms contributed to the genomes and epigenomes of ginseng species complex following polyploidy and speciation and 2) elucidate whether the genes duplicated at different evolutionary stages (ancient vs. recent) and by distinct mechanisms (WGD vs. SSD) have played similar roles in the genome evolution and adaptation. In addition, the functionally important genes identified in this study will have great values for practical applications related to medical and cultural importance of these *Panax* species.

## Results

### Identification of Gene Duplicates Generated by WGD and SSD

To evaluate the impact of the recent WGD (Pg-α) on genome evolution of the three tetraploid *Panax* species, we identified 7,824 orthologous gene pairs (20,061 genes) in the collinear genomic region between the *P. ginseng* and *P. notoginseng* (supplementary table S2, Supplementary Material online). Of these orthologous gene pairs, 1,461 only kept one copy in the two subgenomes of the tetraploid species *P. ginseng* (referred to as singleton gene), and 6,363 were commonly found in the two subgenomes of the tetraploid species *P. ginseng* (doublet gene). Among these doublet genes, 1,958 gene pairs were present in both the diploid and tetraploid species (triplet gene). To further assess the roles of paleopolyploidization in the evolutionary process of the ginseng species complex, we isolated 16,097 genes (referred to as conserved eudicot gene) that showed high collinearity among six modern eudicot species (grape, cacao, carrot, tomato and two ginseng species), 869 of which possessed more than four collinear paralogs in the *P. ginseng* genome (ancient collinear gene) (supplementary table S2, Supplementary Material online). In addition, we identified 1,105–18,812 SSD gene duplicates and 5,108 *Panax*-specific genes in the two *Panax* genomes (supplementary table S2, Supplementary Material online).

### Overall Patterns of Nucleotide Variation, Cytosine Methylation, and Protein Expression

Nucleotide variation patterns of the four *Panax* species were evaluated by calculating the nucleotide diversity (π), genetic divergence (dxy), and selection pressure (*d*_N_/*d*_S_) for each gene. At the genetic level, *P. ginseng* showed relative lower average nucleotide diversity (π = 0.0004) compared with the *P. quinquefolius* (π = 0.0022) and *P. japonicus* (π = 0.0039) (Wilcoxon’s test, all *P* values < 2.2e^−16^) (fig. 1*b*). Likewise, the three tetraploid species also possessed higher variances in terms of the genetic divergence (dxy) and nonsynonymous/synonymous ratio (*d*_N_/*d*_S_) (fig. 1*b*). For cytosine, correlations of the overall cytosine methylation patterns at the intraspecific level (between the two individuals of the same species) (Spearman *R* = 0.92–0.97) are obviously higher than the interspecific comparisons (Spearman *R* = 0.62–0.87) (fig. 1*c*). In particular, the three tetraploid species are more similar to each other (Spearman *R* = 0.83–0.97) than to the diploid species *P. notoginseng* (Spearman *R* = 0.62–0.72). For the proteome data, the generalized linear model (GLM) revealed relatively higher overall expression levels in the three tetraploid species compared with their diploid relative (all *P* values < 2.25e^−8^), whereas higher overall protein-level expressions observed in the three tetraploid species are possibly due to their larger cells relative to the diploid relative. In contrast, no significant differences were observed among the three tetraploid species (all corrected *P* values > 0.01) (fig. 1*d*). The above observations suggest that the four *Panax* species already possessed distinct genetic, epigenetic, and protein-level gene expression patterns at the overall levels.

### Evolutionary Dynamics of the Ancient WGD-Derived Genes

**Fig. 2:**
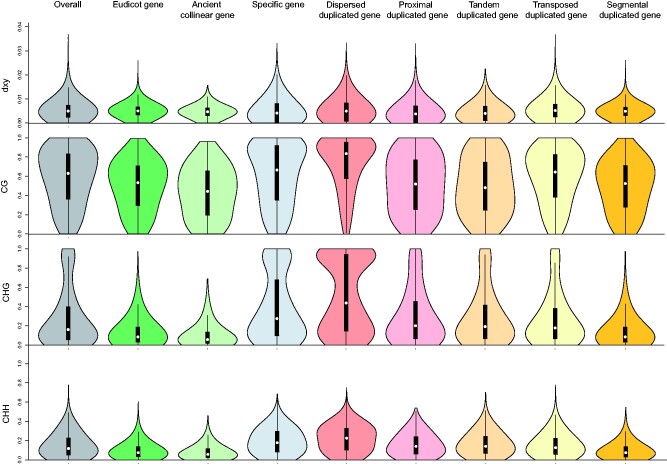
Distribution patterns of the absolute genetic divergence (dxy) and cytosine methylation levels of the overall and different types of duplicated gene in *P. ginseng*. Overall represent the total genes annotated in the *P. ginseng* genome. Eudicot block includes the genes that are collinearly conserved between *P. ginseng* and other four eudicot species (grape, cacao, tomato, and carrot). Ancient collinear gene contains the genes that possess more than four collinear genomic blocks in the *P. ginseng* genome. Specific gene includes the genes that are present in the two *Panax* species and absent in the other four eudicot species. Dispersed, proximal, tandem, transposed, and segmental duplicated genes are the five modes of gene duplicate. Wilcoxon’s two-sample rank-sum test was performed for each comparison.

Long-term evolutionary roles of the paleopolyploidization (i.e., Pg-β) were evaluated by assessing the genetic and epigenetic variation patterns of ancient duplicated genes in the four *Panax* species. At the genetic level, compared with the overall genes, our comparisons among the three tetraploid species revealed that genes within the collinear genomic regions (conserved eudicot gene) exhibited significant lower levels of nucleotide diversity (π) (Wilcoxon’s test, all *P* values < 0.05) and *d*_N_/*d*_S_ ratio (all *P* values < 0.05), especially at the ancient collinear genes (>4 collinear genomic regions) that were duplicated during ancient (i.e., Pg-β) and recent WGDs (Pg-α) (all *P* values < 0.05) (fig. 2 and supplementary fig. S1, Supplementary Material online). In contrast, although significant higher interspecific genetic divergences were observed at the conserved eudicot genes relative to the overall genes (all *P* values < 0.05), all the three tetraploid species possessed opposite pattern at the ancient collinear genes (*P* values = from 2.2e^−16^ to 0.078) (supplementary fig. S1, Supplementary Material online). At the cytosine methylation level, our results revealed that all the four *Panax* species possessed significant lower CG/CHG/CHH methylation level (all *P* values < 0.05) at the conserved eudicot genes relative to the overall genes (fig. 2 and supplementary fig. S1, Supplementary Material online). In particular, this hypomethylation pattern is more evident at the ancient collinear genes across the three types of cytosine sites. These findings suggest that paleopolyploidization (Pg-β) may have played important roles in shaping the genetic and epigenetic patterns of ancient duplicated genes.

### Asymmetric Evolution of the Recent WGD-Derived Genes

**Fig. 3:**
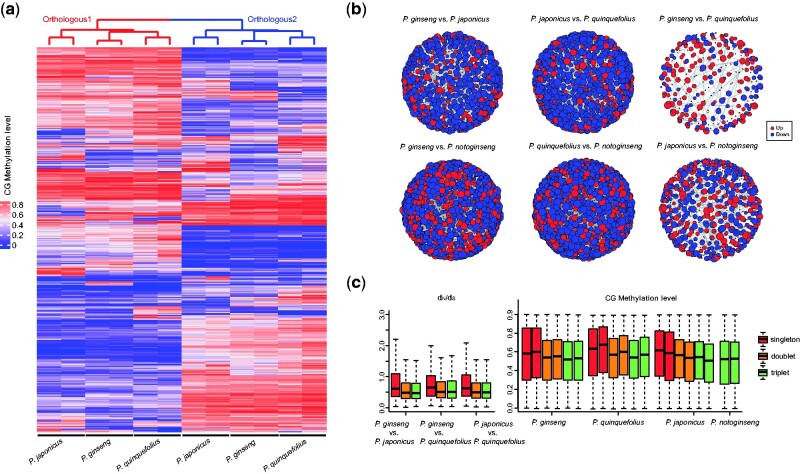
Genetic and epigenetic variation patterns of the recent duplicated genes in the four *Panax* species. (*a*) CG methylation pattern of the recent duplicated gene that showed asymmetrical evolution between the two subgenomes of the three tetraploid species. The red and blue colors on the topologies represent the two subgenomes of the three tetraploid species. Each row represents an orthologous gene pair. (*b*) Protein interaction network of the up- and downregulated proteins of the four *Panax* species. (*c*) Nucleotide variation pattern and CG methylation level of the singleton, doublet, and triplet genes in four *Panax* species.

Impacts of the recent WGD (Pg-α) on the genome and epigenome evolution were estimated by comparing the patterns of nucleotide variation, cytosine methylation, and protein expression between the two subgenomes of the tetraploid species as well as between the diploid and tetraploid species. At the cytosine methylation level, genes showing differential cytosine methylation (referred to as differentially methylated genes, DMGs) were commonly found in all the interspecific comparisons at the CG, CHG, and CHH sites (supplementary tables S3–S5, Supplementary Material online). Among the three tetraploid species, correlations of the overall methylation pattern between the two subgenomes of each species (intraspecific level) (Spearman *R* = 0.64–0.75 for CG; 0.41–0.49 for CHG; 0.42–0.47 for CHH) were more dissimilar compared with those of the same subgenome between different species (interspecific level) (Spearman *R* = 0.80–0.88 for CG; 0.72–0.81 for CHG; 0.78–0.84 for CHH) (supplementary table S6, Supplementary Material online). For example, genes that showed asymmetrical methylation between the two subgenomes were largely consistent across the three tetraploid species (fig. 3*a*). Similar phenomenon was also observed in the population genomic analyses where the correlations of overall nucleotide diversity between the two subgenomes were more dissimilar at the intraspecific level (Spearman *R* = 0.44-0.58) compared with the interspecific comparisons (*R* = 0.79–0.87) (supplementary table S6, Supplementary Material online). These features suggest asymmetric evolution of the recent duplicated genes at both nucleotide variation and cytosine methylation levels in the three tetraploid species. At the proteome level, differentially expressed proteins (DEPs) were commonly observed among the four species (supplementary fig. S2, Supplementary Material online). In the case of *rbcS* gene, two of the five identified paralogous genes showed significantly higher expression levels in the three tetraploid species compared with the diploid species (supplementary table S7, Supplementary Material online). A similar pattern was observed in the protein–protein interaction network where *P. ginseng* and *P. quinquefolius* possess more up- and downregulated proteins compared with the remaining two species (fig. 3*b*).

### Heterogeneous Variation Patterns of the SSD-Derived and *Panax*-Specific Genes

We assessed whether the genes duplicated by SSD mechanisms (dispersed, tandem, proximal, transposed, and segmental) differed in patterns of nucleotide variation (π, dxy, and *d*_N_/*d*_S_) and cytosine methylation (CG/CHG/CHH). Compared with the overall genes, different variances of the nucleotide variation pattern were observed among the five types of SSD genes across the three tetraploid species (most of the comparisons with a *P* value < 0.05) (fig. 2 and supplementary fig. S1, Supplementary Material online). For example, the genes derived from segmental duplication possessed nucleotide variation patterns similar to conserved eudicot genes and ancient collinear genes, whereas relatively higher variances of genetic diversity and divergence were found in the other four types of SSD genes. At the cytosine methylation level, the dispersed gene duplicates are hypermethylated at the three types of cytosine site relative to the overall genes, whereas all the remaining four types of SSD genes are relatively hypomethylated (most of the comparisons with a *P* value < 0.05). In addition to the duplicated genes, we also evaluated the genetic and epigenetic variation patterns of the *Panax*-specific genes. Broadly consistent with the dispersed gene duplicates, high genetic and epigenetic variances were found among the *Panax*-specific genes in the four *Panax* species compared with the overall genes (fig. 2 and supplementary fig. S1, Supplementary Material online). These findings indicate that the five SSD genes and *Panax*-specific genes may have evolved under distinct evolutionary trajectories.

### Biased Fractionation of the Ancient and Recent Duplicated Genes

**Fig. 4:**
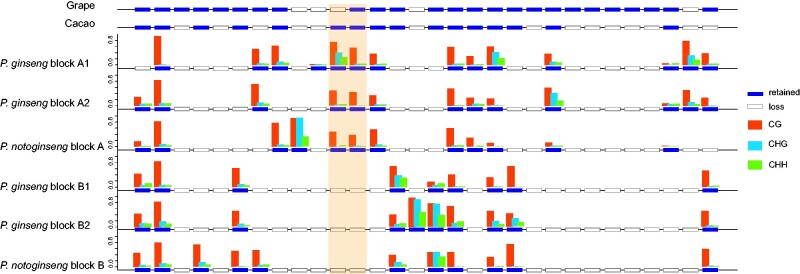
Gene content and cytosine methylation patterns of the collinear genomic block in *P. ginseng* and *P. notoginseng*. The two genes that highlighted by orange color are ancient tandem duplication. Blocks A and B generated by ancient WGD in the two *Panax* species. Blocks A1/A2 and B1/B2 were duplicated during the recent WGD (Pg-α).

The above genome-wide comparisons identified WGD and SSD genes in the four *Panax* species. We then evaluated whether these evolutionary mechanisms played similar roles in shaping the genetic and epigenetic variation patterns of the duplicated genes. Through comparing the genome collinear pattern among the six eudicot species, we identified a conserved genomic block that contained 30 genes in the putative ancestral eudicot genome (fig. 4). Only one syntenic block was present in the grape and cacao genomes; in contrast, two and four orthologous blocks were characterized in the remaining diploid and tetraploid species (carrot, tomato, and ginseng), respectively. Notably, we also identified a tandem duplication gene (highlighted by light pink color) in the block A of all the six eudicot species, indicating that the tandem duplicates originated before the splitting of these species. At the genetic level, our comparative analyses revealed that biased fractionation of the ancestral genes is a common phenomenon in both the diploid and tetraploid *Panax* species. For example, different numbers of retained duplicated gene were observed among the four collinear genomic regions (A1/A2 and B1/B2) in *P. ginseng* (fig. 4). At the epigenetic level, differential methylation of the duplicated genes is also observed at both the WGD and SSD genes across the three types of cytosine site (CG/CHG/CHH).

To further survey the impacts of biased fractionation on the genome and epigenome evolution, we compared the nucleotide variation and cytosine methylation patterns of 7,824 orthologous gene groups in the *P. ginseng* and *P. notoginseng*. Our results revealed that the doublet and triplet genes (no biased fractionation after Pg-α WGD) were not only hypomethylated at the three types of cytosine site but also showed lower variances in terms of the nucleotide variation pattern (π, dxy, and *d*_N_/*d*_S_) compared with the singleton genes (experienced biased fractionation) in all the four *Panax* species (Wilcox’s test, all *P* value < 0.05) (fig. 3*c* and supplementary fig. S3, Supplementary Material online). These findings together indicate that biased fractionation of the ancestral genes is an important mechanism shaping the genetic and epigenetic variation patterns of duplicated gene in the four *Panax* species.

### Functional Enrichment Analyses of the Duplicated and *Panax*-Specific Genes

**Fig. 5:**
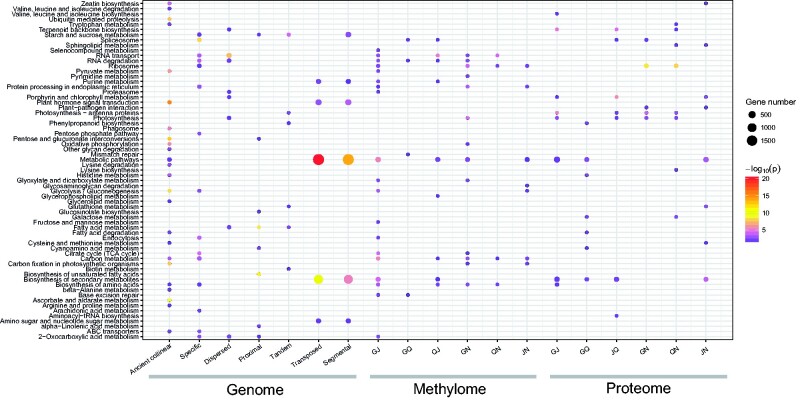
KEGG enrichment analyses of the candidate genes identified from genome, methylome, and proteome data. The characters G, Q, J, and N at the bottom represent the species *P. ginseng*, *P. quinquefolius*, *P. japonicus*, and *P. notoginseng*, respectively. Ancient collinear genes are the duplicates that preserved in the *P. ginseng* genome as collinear (supplementary table S8, Supplementary Material online) genomic blocks. *Panax*-specific genes are the genes present in the *Panax* species only (supplementary table S10, Supplementary Material online).

The above approaches identified candidate genes from the genome-wide genetic and epigenetic comparisons. We then examined whether these candidate genes are associated with specific traits of the four *Panax* species. At the genetic level, although duplicated genes generated by WGD and SSD mechanisms are significantly (corrected *P* value < 0.05) enriched in diverse categories, some functional pathways related to plant growth (i.e., plant hormone signal transduction) and photosynthesis (i.e., carbon fixation in photosynthetic organisms) were commonly identified in ancient collinear, segmental, and tandem duplication genes (fig. 5 and supplementary tables S8 and S9, Supplementary Material online). In contrast, the *Panax*-specific genes are mainly associated with basic cellular activities, such as citrate cycle, carbon metabolism, and spliceosome (supplementary table S10, Supplementary Material online). At the methylatomic and proteomic levels, candidate genes characterized from the interspecific comparisons are also enriched in diverse functionally important pathways (fig. 5 and supplementary tables S7 and S12–S15, Supplementary Material online). Notably, partial of the DEPs and DMGs identified between the tetraploid and diploid species are also enriched in photosynthesis-related pathways, including photosynthesis-antenna proteins, carbon fixation in photosynthetic organisms and porphyrin and chlorophyll metabolism.

## Discussion

### Asymmetrical Evolution of the Ginseng Species Complex Following Recent Polyploidy and Speciation

Polyploidy has long been recognized as an important driving evolutionary force in promoting plant adaptation and diversification (Fawcett et al. 2009; Vanneste et al. 2014; Van de Peer et al. 2017; Landis et al. 2018). Recent investigations from diverse polyploid species have confirmed the genetic and epigenetic consequences of polyploidy in the short- and long-term evolutionary processes (Cheng et al. 2018; Wendel et al. 2018). In *Panax*, we have proposed that two rounds of WGD together with geographic and ecological isolations promoted the genome evolution and species diversification (Shi et al. 2015). In this study, we focused on how the genes duplicated by recent WGD contributed to the genome evolution and adaptation of ginseng species complex following polyploidy and speciation. It has been documented that genetic and epigenetic asymmetry of the duplicated genomes is a common feature in many neopolyploid species (Wendel et al. 2016; Cheng et al. 2018). In cotton, for example, asymmetrical pattern of homoelogous expression at transcriptional and protein levels is observed in both synthesized and natural polyploids (Hu et al. 2013, 2015, 2016; Wendel et al. 2018). Here our genome-wide comparisons also revealed that genetic and epigenetic asymmetry are commonly observed at the recent WGD genes of the four *Panax* species, whereas they possessed distinct patterns of nucleotide variation, cytosine methylation, and protein-level expression at the overall level. In particular, our results indicate that partial of the duplicated genes showing asymmetric pattern are largely consistent across the three tetraploid species. Given that the ginseng species complex was formed through a recent tetraploidization event (1.6–3.3 Ma) (Choi et al. 2013; Shi et al. 2015), it is likely that asymmetric pattern of the recent WGD genes, at least partially, was established in the common ancestral genome after the allotetraploidization event (Pg-α) and then maintained in the three descendant tetraploid species postspeciation.

As a typical pattern of asymmetric evolution, biased fractionation of the redundant genes and DNA sequences is widely reported in diverse polyploid species (Wendel et al. 2016). In wheat, high frequency of aneuploidy and extensive genomic and chromosomal variations are observed in the resynthesized polyploids mimicking natural polyploid wheats (Zhang, Bian, Gou, Dong, et al. 2013; Zhang, Bian, Gou, Zhu, et al. 2013; Bian et al. 2018). In the ginseng species complex, biased retention of duplicated genes was also observed at both collinear ancient gene pairs (derived from ancient Pg-β and earlier WGD) and orthologous gene pairs (derived from recent Pg-α WGD). Theoretically, duplicated genes are expected to undergo relaxed purifying selection, which will eventually result in the increasing of genetic diversity and novelty (Cheng et al. 2018). However, our results demonstrate that the singleton genes not only accumulated more genetic polymorphisms (π) but also showed high variances in terms of the selection pressure (*d*_N_/*d*_S_) and genetic divergence (dxy) compared with the doublet and triplet genes in all the three tetraploid species. In contrast, hypermethylation of the singleton gene observed in the ginseng species complex is broadly consistent with previously investigated species, such as potato, tomato, and common bean (Wang et al. 2018). Collectively, these genomic and epigenomic features suggest that asymmetric evolution of duplicated genes may have played important roles in shaping the genomes and epigenomes of the ginseng species complex following polyploidy and speciation.

Evolutionary success of polyploid species is mainly attributed to their high adaptability to diverse ecological environments (Hegarty and Hiscock 2008; Van de Peer et al. 2017). Polyploid species have been reported to be more common in extreme environments compared with their low ploidy relatives (Grant 1981; Brochmann et al. 2004). In wild yarrow, hexaploid populations show higher fitness advantage over tetraploids in dune habitats relative to the mesic grassland, with genome duplication per se explaining about 70% of the fitness advantages (Ramsey 2011). A similar phenomenon is also evident in the genus *Panax* where high ploidy species often occupy ecological niches of high latitude and altitude (Shi et al. 2015; Zuo et al. 2017). Here, our study demonstrated that candidate genes identified in the genetic and epigenetic comparisons were associated with functionally important traits, such as photosynthesis, plant–pathogen interaction, and basic cellular activities. For example, all Panax species are shade-demanding perennial herbs inhabiting cool shaded environments (Liu and Xiao 1992; Chen et al. 2014, 2016). It has been proposed that the chloroplast (i.e., *rbcL*) and photosynthesis-related nuclear genes are potentially associated with the adaptive evolution of *Panax* species (Li et al. 2017; Jiang et al. 2018). Here, our results further revealed that the nuclear *rbcS* gene encoding the small unit of rubisco (a rate-limiting enzyme of photosynthesis) is significantly upregulated at the protein expression level in the three tetraploid species compared with their diploid relative. Given that the cytonuclear coevolution of the large (*rbcL*) and small (*rbcS*) rubisco units has played important roles in the genome evolution of polyploid species (Gong et al. 2012), it is likely that functional enrichment of the photosynthesis-related pathways may have conferred fitness advantage to the ginseng species complex.

### Evolutionary Contribution of the Ancient WGD Gene Duplicates

Ancient WGD is a widespread phenomenon in all extant angiosperm plants (Jiao et al. 2011; Renny‐Byfield and Wendel 2014; Soltis PS and Soltis DE 2016). Although ancient polyploidy was recognized as early as 70 years ago (Stebbins 1950), its roles in the phenotypic novelty and species diversification are still not fully understood. Unravelling the evolutionary fates of ancient duplicated genes may be central to understanding how paleopolyploidization has shaped genome evolution of extant plant species (McGrath and Lynch 2012; Magadum et al. 2013). In Buckler mustard, for example, biased fractionation of the ancient and recent duplicated genes resulted in the recruitment of stress-responding genes (Geiser et al. 2016).

In this study, we investigated whether the gene duplicates derived from ancient WGDs have contributed to genome evolution of the four *Panax* species. We found that collinear ancient genes (mainly derived from Pg-β and earlier WGDs) are not only genetically conserved (dxy and π) and under higher purifying selection pressure (*d*_N_/*d*_S_) but also show hypomethylation at all three cytosine contexts. More importantly, some of the gene duplicates were enriched in categories related to functionally important traits, such as plant hormone signal transduction and carbon fixation in photosynthetic pathway. Plant hormones are a group of structurally unrelated small molecules that regulate plant growth and mediate responses to both biotic and abiotic stresses (Santner et al. 2009; Santner and Estelle 2009). Likewise, carbon fixation or assimilation is the key step in photosynthesis that multiple enzymes (e.g., Rubisco) involved in the Calvin cycle work together to convert inorganic carbon to organic compounds (Ducat and Silver 2012). Multiple duplicated copies of these functionally important genes are potentially associated with the ecological adaptation of *Panax* species to dime light environments. Similar phenomena were also observed in other plant species where the retention of gene duplicates, such as *AUS/IAA* family of auxin response regulators and glucosinolate synthetic genes, has conferred fitness advantage to the plant species (Remington et al. 2004; Edger et al. 2015). In light of these findings, our results in *Panax* suggest that the fitness advantage (i.e., efficiency of photosynthesis and plant growth) created during the distant ancient WGD is further strengthened by the more recent WGD.

### Distinct Evolutionary Mechanisms but Playing Similar Roles in Shaping the Genome Evolution

Our genetic and epigenetic comparisons revealed important roles of the ancient and recent WGD-derived duplicate genes following polyploidy and speciation. We then evaluated whether the duplicate genes generated by SSD mechanism have played similar roles in shaping the genome evolution of ginseng species complex. Previous studies have demonstrated that duplicate genes generated by SSD and WGD mechanisms often show different evolutionary dynamics and functional features (Carretero-Paulet and Fares 2012; Rensing 2014; Li et al. 2016; Qiao et al. 2019). For example, the numbers of ancient WGD-derived duplicate genes tended to decline exponentially in modern plant genomes compared to those generated by SSD mechanisms (i.e., duplication- and dispersed duplication-derived genes) (Qiao et al. 2019). In particular, the tandem duplication- and proximal duplication-derived genes experienced stronger selective pressure than those genes generated by other SSD modes and evolved toward biased functional roles related to plant self-defense (Qiao et al. 2019). Broadly consistent with previous observations, our analyses also revealed distinct genetic and epigenetic variation patterns among the different types of duplicate genes. For example, compared with the overall genes, the WGD-derived duplicate genes (i.e., ancient collinear gene) are not only hypomethylated at the cytosine sites (CG/CHG/CHH) but also showed lower variances in term of the nucleotide variation pattern (π, dxy, and *d*_N_/*d*_S_). However, opposite patterns of the cytosine methylation and nucleotide variation were observed in some SSD-derived duplicate genes (i.e., dispersal duplication-derived gene). In addition, our results revealed that genes duplicated by distinct mechanisms (WGD vs. SSD) and at different evolutionary stages (ancient vs. recent) are enriched in diverse functional categories. Nevertheless, we found that genes involving in plant growth and photosynthesis were not only characterized in the WGD- (i.e., ancient collinear gene) and SSD-derived duplicate genes (i.e., tandem duplication-derived gene) but also identified at both the genetic (*Panax*-specific genes) and epigenetic (i.e., variation in DNA methylation and DEPs) comparisons. Together, these findings suggest that genes derived from different duplication mechanisms (SSD vs. WGD) and at distinct evolutionary stages (ancient vs. recent) may have played distinct but relaying evolutionary roles in the polyploidization and speciation processes of the ginseng species complex.

## Materials and Methods

### Plant Materials, DNA, and Protein Extraction

A total of 104 accessions from four *Panax* species were used in this study, including three tetraploid species (*P. ginseng*, *P. quinquefolius*, and *P. japonicus*) and one diploid species (*P. notoginseng*) (supplementary table S1, Supplementary Material online). In addition, eight and 12 representative accessions were selected from the four species to assess cytosine methylation and protein expression patterns, respectively. All the selected *Panax* accessions were grown in green house under the same conditions (18 °C/12 h, 24 °C/12 h). Genomic DNA for whole genome and methylome sequencing was extracted from silica gel dried leaves and fresh main root using TianGen plant genomic DNA Kit (TianGen, Tianjin, China), respectively. Total proteins of the selected 12 accessions (three accessions per species) were extracted from fresh mature leaves using Lysis Buffer (50 mM Tris-base, 8 M Urea, and 1% SDS). Peptide samples were generated by digesting the proteome for each accession using Trypsin Gold (Promega, Madison, WI).

### Whole Genome Resequencing, Methylome, and Protein Expression

Whole genome and methylome sequencing were performed by NovoGene (Tianjin, China) using Illumina X-ten platform (Illumina, CA). Clean reads of the whole genome data were mapped onto the reference genome of *P. ginseng* using BWA (Li and Durbin 2010) using the parameter set as “bwa men -n 0.05.” Raw assemblies of these *Panax* accessions were realigned to corresponding reference genomes using Genome Analysis Toolkit (GATK) IndelRealigner version 2.6 (McKenna et al. 2010). Single nucleotide polymorphisms (SNPs) and insertions/deletions (INDELs) were identified using SAMtools (Li et al. 2009) with the parameter “mpileup -Dsugf -C 50 -q 30 -Q 20” and “bcftools view -Ncvg.” The raw variants were further filtered using Perl script with the threshold “mapping quality (MQ) > 30, read depth (RD) > 3.” Raw short reads of the eight methylome data sets were trimmed using the program TrimGalore (www.bioinformatics.babraham.ac.uk/projects/trim_galore) with the default parameters. Filtered clean reads of the four species were mapped onto the reference genomes of *P. ginseng* and *P. notoginseng* using Bismark (Krueger and Andrews 2011), respectively. Only the CG/CHG/CHH sites with total read depth (methylated + unmethylated) higher than ten across all the eight accessions were included in subsequent analyses. Quality control of the whole genome resequencing and methylome data were detailed in supplementary notes, Supplementary Material online. For proteome data, the *Panax* protein library was constructed by combining the nonredundant protein data sets of the *P. ginseng* and *P. notoginseng* (Zhang et al. 2017; Kim et al. 2018). Raw peptide data generated in this study were applied to search against the *Panax* proteome library and quantified using the program Proteome Discoverer (Thermo Fisher Scientific, KS). A total of 49,745 peptides corresponding to 9,492 proteins were identified in the 12 *Panax* accessions, 8,009 (84.5%) of which were successfully annotated in the GO/KEGG/COG database.

### Nucleotide Variation Pattern and Natural Selection

Variants (SNPs and INDELs) of the 104 *Panax* accessions were generated based on the reference genome of *P. ginseng* (Kim et al. 2018). Previous study has identified two paralogous groups (subgenomes) in the reference genome. To this end, population genomic analyses were performed for the overall genes and two paralogous groups separately (see details in supplementary notes, Supplementary Material online). To evaluate whether genes duplicated by WGD and SSD mechanisms contributed to the genome evolution and adaptation following polyploidy and speciation, we calculated nucleotide diversity (π) and genetic divergence (dxy) for each gene of the three tetraploid species using VCFtools (Danecek et al. 2011). Present and absent of gene in the *P. quinquefolius* and *P. japonicus* were determined by estimating the average read depth (supplementary notes, Supplementary Material online). Ratio between nonsynonymous (*d*_N_) and synonymous (*d*_S_) mutation rate was estimated for each gene using PAML (Yang 2007).

### DMGs and Expressed Proteins

Interspecific DMGs were identified using Cochran–Mantel–Haenszel test. Only the genes that are fully mapped for each species pair were employed to perform the Cochran–Mantel–Haenszel test. To minimize the false positives that might be caused by the statistical method, we filtered the raw DMGs according to the following criteria: 1) intraspecific methylation difference smaller than 10% (fisher exact test, *P* value < 0.01), 2) interspecific methylation difference greater than 50%, and 3) more than 10% of the gene body region are statistically significant (corrected *P* value < 0.01). As the overall methylation levels of the three types of cytosine site are relatively high in the four *Panax* species, we thus reexamined methylation patterns of the eight samples using different strategies (see details in supplementary notes, Supplementary Material online). In addition, DEPs were determined by the program DESeq2 (Love et al. 2014). Overall patterns of the DMGs and DEPs were visualized using pheatmap (Kolde 2015). Functional enrichment of these DMGs and DEPs was performed using KEGG (Kanehisa and Goto 2000). In addition, interaction network of the DEPs was visualized using Cytoscape (Shannon et al. 2003) based on the StringDB protein database. Only these protein pairs with score >150 were included in the protein–protein interaction network.

### Identification of the WGD and SSD Genes

Duplicated genes generated by WGD and SSD mechanisms were identified in both the tetraploid *P. ginseng* and diploid *P. notoginseng*. Gene duplicates derived from recent WGD (Pg-α) were determined by identification of the collinear genomic regions between the tetraploid *P. ginseng* and diploid *P. notoginseng* using MCScanX (Wang, Tang, et al. 2012). Likewise, gene duplicates generated by ancient WGDs (i.e., Pg-β) were characterized by comparing the genome structure between the *P. ginseng* and other four eudicot species (tomato, grape, cacao, and carrot). Grape and cacao are the most conserved genomes among all extant eudicot plants and which did not experience additional paleopolyploidization events after their splitting from the common eudicot ancestor (Murat et al. 2017). Tomato and carrot are the closest species with assembled genomes to the *Panax* species. Given that *P. ginseng* has experienced additional rounds of WGD (i.e., Pg-α and Pg-β) after the eudicot-shared γ triplication, these genes that possessed more than four collinear homologous copies in the *P. ginseng* genome relative to grape are defined as ancient collinear genes. In addition, we also identified five types of SSD genes from the *P. ginseng* genome using DupGenFinder (Qiao et al. 2019), including dispersed, proximal, tandem, transposed, and segmental gene duplicates. As a supplementary, we characterized gene families from the two *Panax* and other four selected eudicot species using OrthoMCL (Li et al. 2003). These gene families only present in either of the two *Panax* species are defined as *Panax*-specific genes.

## Supplementary Material

Supplementary data are available at *Genome Biology and Evolution* online.

## Supplementary Material

evab051_Supplementary_Data
