# Supplementary material for: Evolutionary Contribution of Duplicated Genes to Genome Evolution in the Ginseng Species Complex
Source: Genome Biol Evol. 2021 Mar 13;13(5):evab051. doi: 10.1093/gbe/evab051 (PMC8103499; doi:10.1093/gbe/evab051)
Supplement: evab051_Supplementary_Data [file evab051_supplementary_data.doc.zip › Supplementary Notes.docx]

**Supplementary Notes**

**Quality control and population genomic analyses of the whole genome resequencing data**

Whole genome sequences of the 104 *Panax* accessions were mapped onto the reference genome of *Panax ginseng* (Kim *et al*., 2018). More than 83.5% of these *Panax* samples show high mapping rate (>90%), particularly that almost all of the *P. quinquefolius* and *P. notoginseng* samples possess very high mapping rate (>98%) (**Table S1**). It suggests that the reference genome of *P. ginseng* is suitable to perform population genomic analyses. In addition, Kim *et al*. (2018) identified two groups of paralogous scaffolds in the *P. ginseng* genome. Each of the two paralogous groups represents a subgenome of the tetraploid species *P. ginseng*. To this end, population genomic analyses were performed for the whole genome and the two paralogous groups, respectively. Absolute genetic divergence and nucleotide diversity were not calculated for *P. notoginseng* because only two accessions were used in this study. Present and absent of gene in the *P. quinquefolius* and *P. japonicus* were determined by estimating the average read depth. These genes that more than 50% genic region are not covered by high quality reads (mapping quality (MQ) > 30, read depth (RD) > 3) were defined as absent in the two tetraploid species. By comparing the average read depth of the 12,899 orthologous genes, only the *P. japonicus* contained 299 (2.3%) low depth genes that are supposed to be lost after the divergent from the common ancestor. We believe that our results are able to reflect the genetic background and evolutionary histories of the four *Panax* species.

**Quality control and methylation pattern assessments of the methylome data**

We note that overall cytosine methylation levels of the four *Panax* species are relatively higher compared to most of the other flowering plants (Niederhuth *et al*., 2016; Takuno *et al*., 2016). To confirm that high cytosine methylation level observed in the *Panax* species was not generated by the experimental technologies, we employed different strategies to examine the methylome data.

(1) *Impacts of tissue-specific, mapping rate, BS conversion rate and library construction strategy on the cytosine methylation levels*

Methylome data of the *P. japonicus* accession Z4 was generated from both the main root and mature leaf, respectively (**Table S15**). Our comparisons clearly revealed that overall methylation levels of the main root (CG, 78.6%; CHG, 70.1%; CHH, 30.6%) are apparently higher than mature leaf (CG, 69.9%; CHG, 52.5%; CHH, 16.2%). However, we noted that the mature leaf shows lower BS conversion rate (95.86%). To this end, a technical replicate of the same sample of mature leaf was sequenced. Although the overall cytosine methylation level of the technical replicate is increased (CG, 76.00%; CHG, 64.00%; CHH, 20.70%), it is still similar to that of the *Beta vulgaris* (CG, 92.5%; CHG, 81.2%; CHH, 18.8%) (Niederhuth *et al*., 2016). We also employed a minimal library construction strategy to examine the cytosine methylation level of the same sample of mature leaf. Although the overall cytosine methylation level is decreased, we found that the mapping rate (14.9%) is also dropped compared to the normal library strategy (28.77-32.49%). Given that morphological differences of the four *Panax* species are mainly identified in the root tissue and the minimal library strategy cannot generate enough methylome data for each library, we generated all methylome data from main root of the four *Panax* species using the normal library strategy.

(2) *Influences of reference genome on the cytosine methylation level*

While methylome data was generated from the four *Panax* species, reference genomes are only available for the *P*. *ginseng* and *P. notoginseng*. To test whether reference genomes can increase the mapping rate, a new reference genome of *P. japonicus* was generated by replacing the *P. ginseng* genome using genetic variants identified in the resequencing data of *P. japonicus*. Filtered methylome data of the accession JI3 was mapped onto to original *P. ginseng* and replaced reference genomes, respectively. Our comparison revealed that relatively higher mapping rate was observed at the replaced reference genome (41.8%) compared to the original *P. ginseng* genome (30.18%). Interestingly, the overall methylation levels increased slightly at the replaced reference genome (**Table S15**). Given that the three tetraploid species were formed through a recent tetraploidization event, subsequent methylation analyses of the two species (*P. japonicus* and *P. quienquefolius*) were performed using the replaced reference genomes. Likewise, methyome data of *P. notoginseng* was mapped onto both the *P. notoginseng* and replaced reference genomes, respectively. In addition, overall methylation levels of the four species were also reexamined using the minimal library strategy. Although all the four *Panax* species show relatively higher mapping rates and methylation levels using the normal library strategy, genome-wide methylation patterns are highly correlated between the two library strategies across the four species (Spearman’s correlation, R = 0.81-0.85, *p* values < 0.01) (**Table S15**). These attributes suggest that the strategy used to generate methylome data should not result in the data bias.

(3) *High filtering criteria* *were employed to identify the DMGs*

To further minimize the false positives that caused by statistic methods, we employed high filtering criteria to identify the differentially methylated genes (DMGs) among the four *Panax* species (see details in section **Materials and** **Methods**). In brief, only the genes meet the following three criteria are defined as DMGs: *i*) no significantly different between accessions within the same species (fisher exact test, *p* value < 0.01); *ii*) high methylation divergence (> 50%) between species; *iii*) more than 10% of the gene body regions are differentially methylated (CMH test, corrected *p* value < 0.01). Taken together, we believe that overall methylation patterns and DMGs identified are reliable.

**Reference**

**Kim N.H., Jayakodi M., Lee S.C., Choi B.S., Jang W**., et al., 2018. Genome and evolution of the shade-requiring medicinal herb *Panax ginseng*. *Plant Biotechnology Journal* 16:1904-1917.

**Niederhuth C.E., Bewick A.J., Ji L., Alabady M.S., Kim K.D**., et al. 2016. Widespread natural vaiation of DNA methylation within angiosperms. *Genome Biology* 17:194.

**Takuno S., Ran J.H., Gaut B.S**. 2016. Evolutionary patterns of genic DNA methylation vary across land plants. *Nature Plants* 2:15222.

**SUPPORTING INFORMATION**

**Supplementary Figures**

**Figure S1.** Nucleotide variation patterns (π, d_N_/d_S_ and dxy) (A) and cytosine methylation levels at CG, CHG and CHH sites (B) of the total and duplicated genes in the four *Panax* species. Wilcoxon's two-sample rank-sum test was performed for each comparison. Only the non-significant comparisons (*p* value > 0.05) are shown.

**Figure S2**. Heatmap of the differentially expressed proteins (DEPs) among the four *Panax* species.

**Figure S3.** Nucleotide variation and cytosine methylation patterns of the singleton, doublet and triplet genes in four *Panax* species.

**Supplementary Tables**

**Table S1**. Mapping rates of the whole genome sequences of the 103 *Panax* accessions.

**Table S2**. Identification of the gene duplicates in the *Panax ginseng* and *Panax notoginseng*.

**Table S3**. Differentially methylated genes (DMGs) at the CG site of the four *Panax* species. Each DMG contains multiple 100 bp differentially methylated bins. Only these genes that more than 10% of the total length are differential methylated (CMH test, *p* value < 0.01) are defined as DMG. Odds ratio means the ratio of methylation between the two species.

**Table S4**. Differentially methylated genes (DMGs) at the CHG site among the four *Panax* species. Definition of the DMGs is the same as CG site.

**Table S5**. Differentially methylated genes (DMGs) at the CHH sites among the four *Panax* species. Definition of the DMGs is the same as CG site.

**Table S6.** Correlations of the overall genetic and epigenetic patterns of the three tetraploid species at both the intra- and inter-specific levels.

**Table S7**. KEGG enrichment of the differentially expressed proteins among the four *Panax* species.

**Table S8**. KEGG enrichment of the ancient collinear gene in *Panax ginseng*.

**Table S9**. KEGG enrichment of the singleton, dispersed, proximal, tandem, and segmental duplicated genes.

**Table S10**. KEGG enrichment of the *Panax*-specific genes in *Panax ginseng*.

**Table S11**. KEGG enrichment analysis of the differential methylated genes at CG site among the four *Panax* species.

**Table S12**. KEGG enrichment analysis of the differential methylated genes at CHG site among the four *Panax* species.

**Table S13**. KEGG enrichment analysis of the differential methylated genes at CHH site among the four *Panax* species.

**Table S14**. KEGG enrichment of the differential methylated genes at all cytosine site among the four *Panax* species.

**Table S15**. Overall DNA methylation levels of the four *Panax* species based on different strategies.
