# Supplementary figures and images for: Evolutionary Contribution of Duplicated Genes to Genome Evolution in the Ginseng Species Complex
Source: Genome Biol Evol. 2021 Mar 13;13(5):evab051. doi: 10.1093/gbe/evab051 (PMC8103499; doi:10.1093/gbe/evab051)

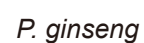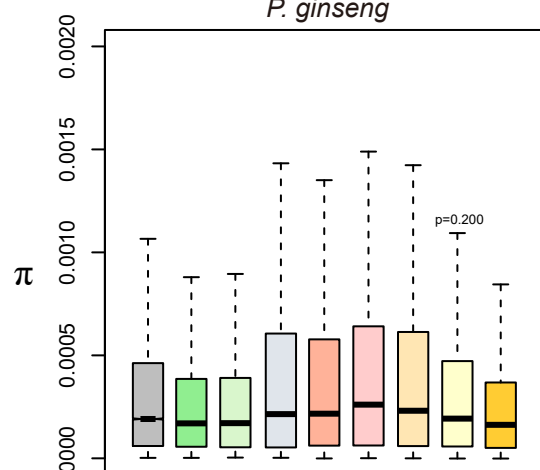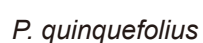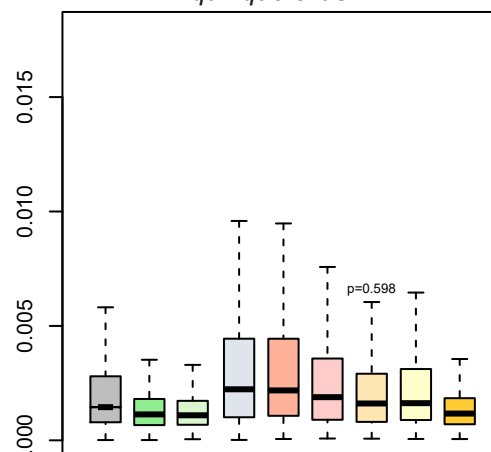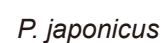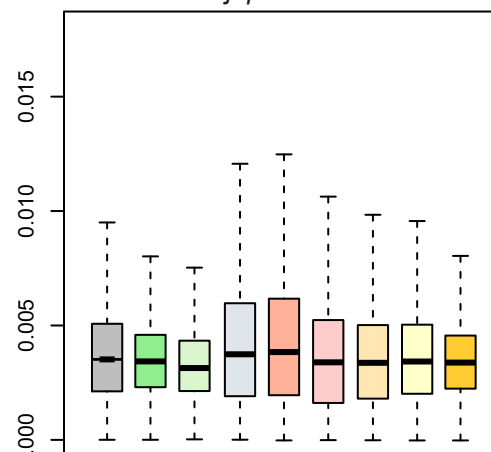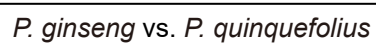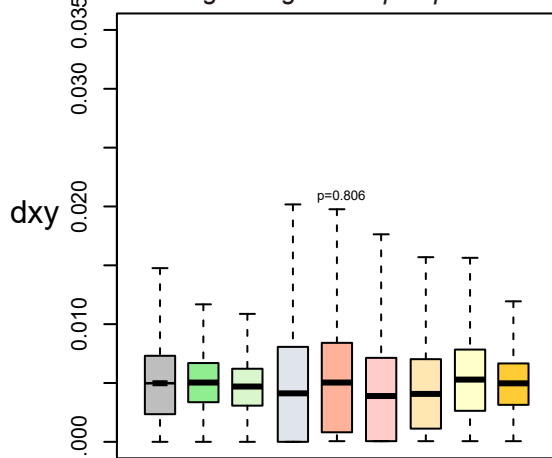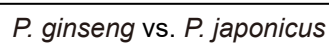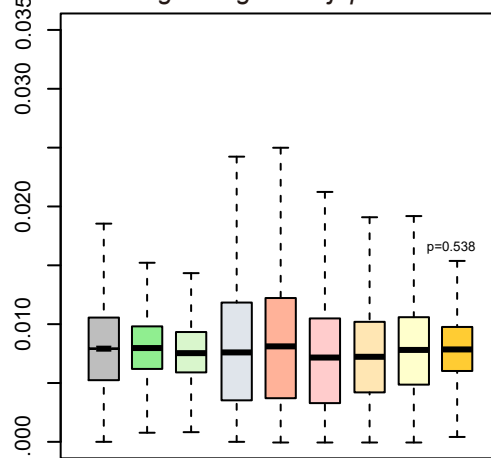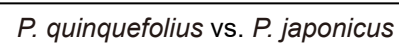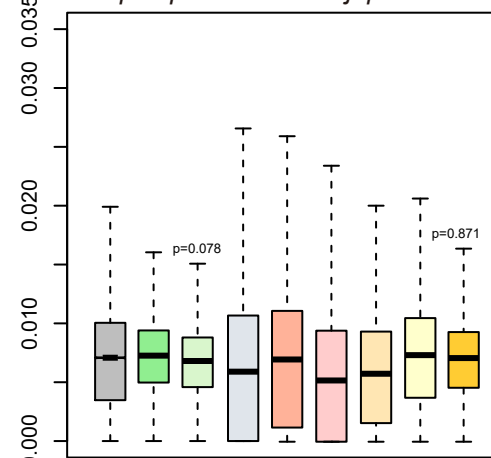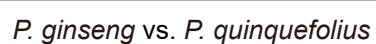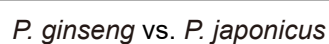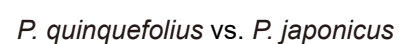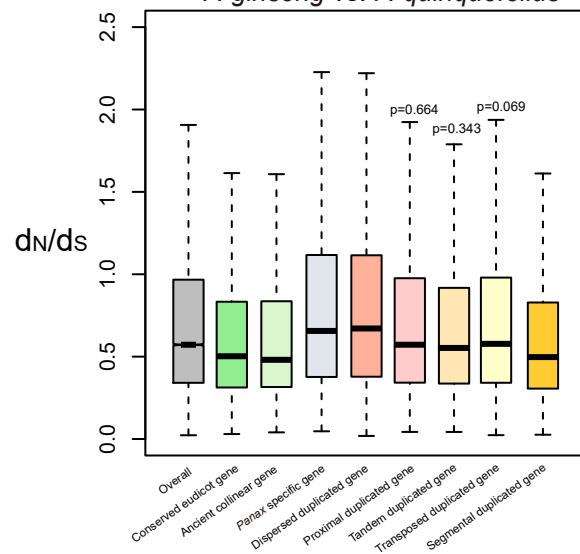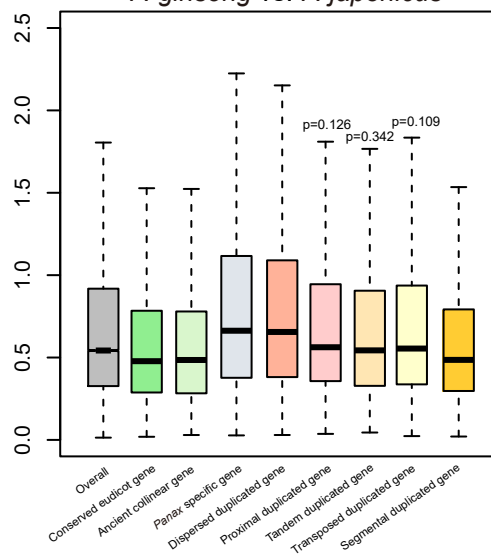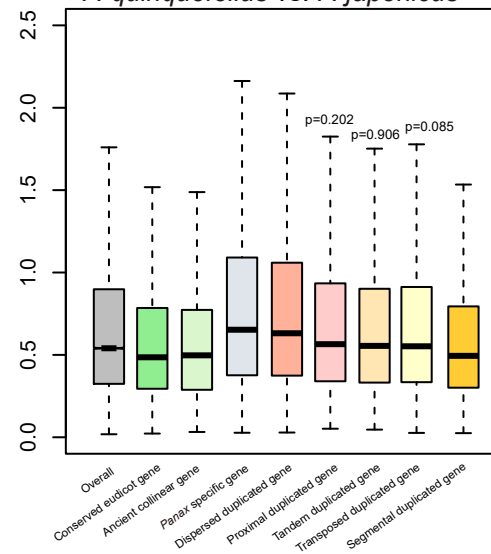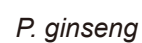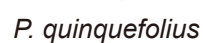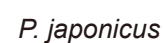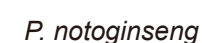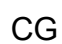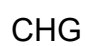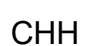

Supplement: evab051_Supplementary_Data [file evab051_supplementary_data.doc.zip › Figure S1.pdf]

Differential expressed proteins

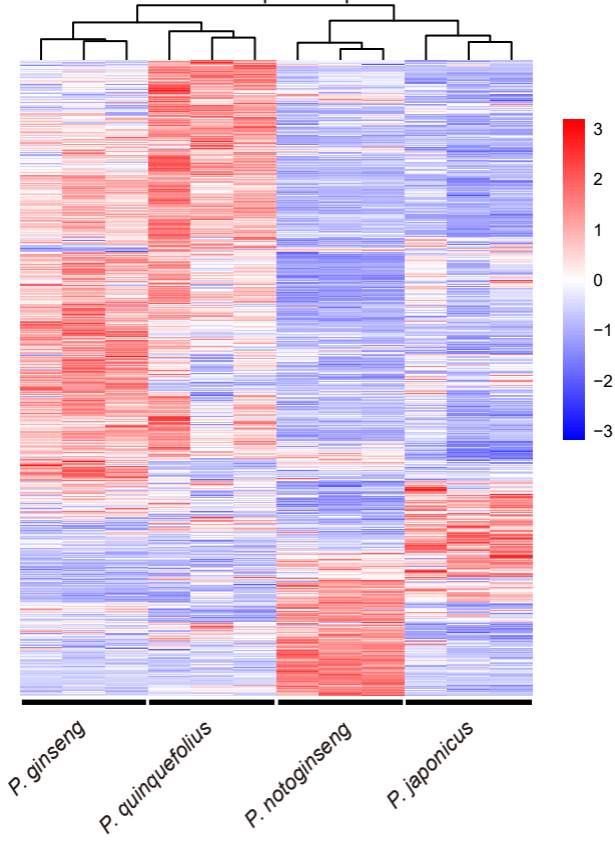

Supplement: evab051_Supplementary_Data [file evab051_supplementary_data.doc.zip › Figure S2.pdf]

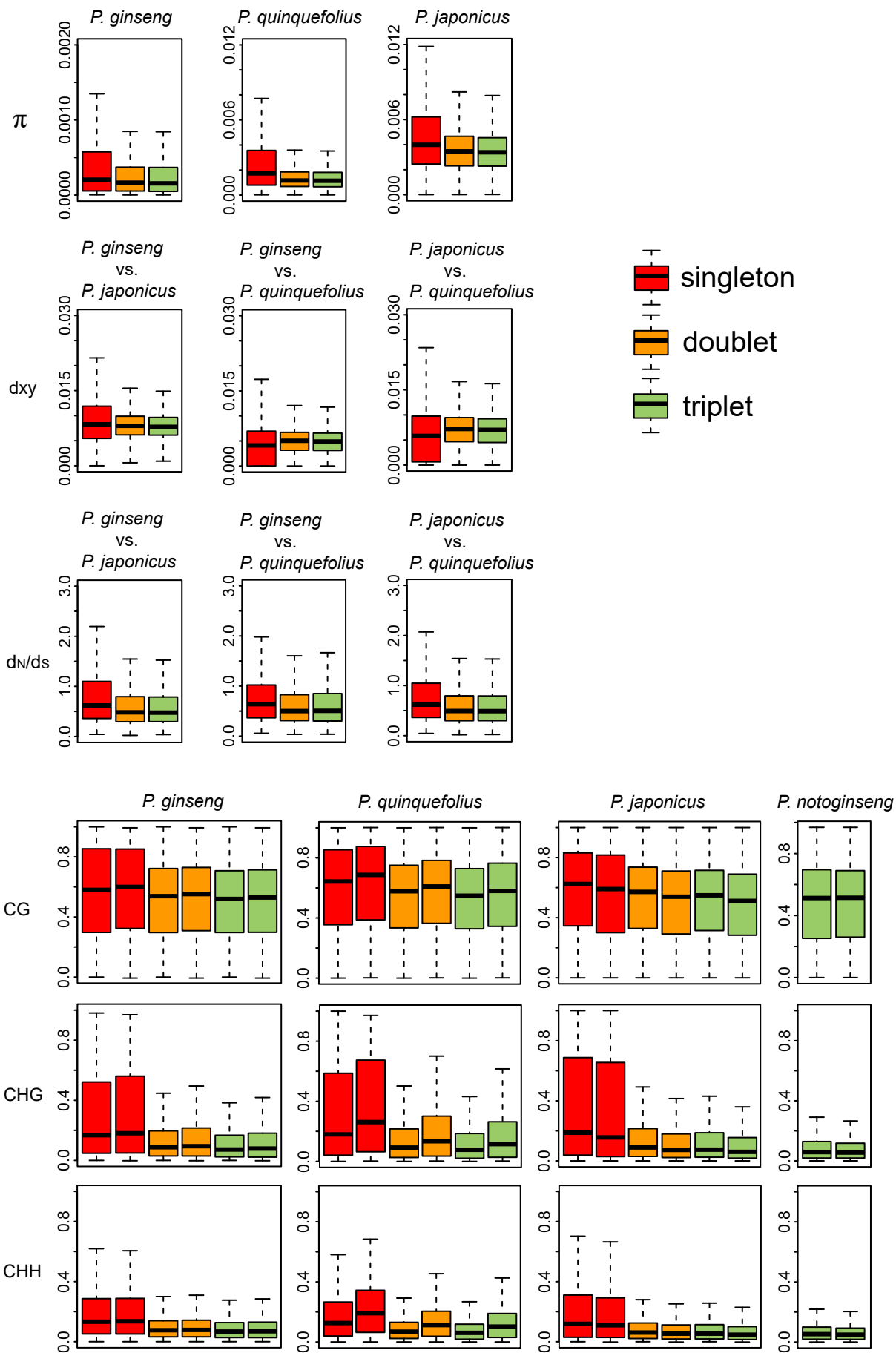

Supplement: evab051_Supplementary_Data [file evab051_supplementary_data.doc.zip › Figure S3.pdf]
